# Saponins from *Momordica charantia* exert hypoglycemic effect in diabetic mice by multiple pathways

**DOI:** 10.1002/fsn3.3682

**Published:** 2023-09-18

**Authors:** Yuanyuan Deng, Yan Zhang, Guang Liu, Pengfei Zhou, Ping Li, Zhihao Zhao, Ruifen Zhang, Xiaojun Tang, Zhangying Wang, Zhencheng Wei, Mingwei Zhang

**Affiliations:** ^1^ Sericultural & Agri‐Food Research Institute, Guangdong Academy of Agricultural Sciences/Key Laboratory of Functional Foods Ministry of Agriculture and Rural Affairs/Guangdong Key Laboratory of Agricultural Products Processing Guangzhou PR China; ^2^ Crops Research Institute Guangdong Academy of Agricultural Sciences/Key Laboratory of Crop Genetic Improvement of Guangdong Province Guangzhou China

**Keywords:** hypoglycemic effects, mice, *Momordica charantia*, pathways, saponin

## Abstract

The antidiabetic activity of saponins extracted from *Momordica charantia* (MCS) on streptozotocin‐induced diabetic mice was investigated in order to elucidate the mechanism of MCS for exerting hypoglycemic effects. Saponins were first extracted from *M. charantia* L. and their composition was analyzed. The diabetic Kunming mice were fed low‐dose saponins from *M. charantia* L. and high‐dose MCS, using normal mice and diabetic mice as controls. Body weight, blood glucose level, oral glucose tolerance, serum C‐peptide level, hepatic antioxidant capacity, hepatic glycogen and hexokinase in liver tissues, serum blood lipid level, and alpha‐glucosidase activity in small intestines were measured, and microstructure of pancreatic islet was analyzed. The results showed that the total content of seven triterpenoid compounds in MCS was 18.24 μg/mg, with Momordicoside K having the highest content at 11.66 μg/mg. Diabetic mice treated with MCS at 100 and 200 mg/kg body weight daily for 30 days showed a maximum glucose reduction (*p* < .05) of 12.63% and 26.47%, respectively. MCS significantly decreased levels of postprandial hyperglycemia, serum lipid, α‐glucosidase activity, and liver malondialdehyde. Additionally, levels of serum C‐peptide and liver glycogen, as well as hexokinase and antioxidant enzyme activity, were significantly increased compared to the diabetic control groups. Histopathological results showed that MCS markedly reduced degenerative changes in islet β‐cells. It is concluded that MCS exerts antidiabetic effects by improved hypoglycemic, hypolipidemic, and antioxidant effects, increased hexokinase activity and glycogen synthesis, and enhanced reparative effects on the histological architecture and insulin secretion function of the pancreas.

## Graphical abstract

*Momordica charantia* saponin (MCS) significantly decreased levels of postprandial hyperglycemia, serum lipid, a‐glucosidase activity, and liver malondialdehyde. Additionally, levels of serum C‐peptide and liver glycogen, as well as hexokinase and antioxidant enzyme activity, were significantly increased compared to the diabetic control groups. Histopathological results showed that MCS markedly reduced degenerative changes in islet ß‐cells.

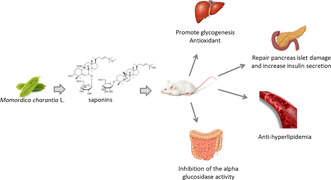

## 1 INTRODUCTION

According to the International Diabetes Federation, the global population of people with diabetes reached 537 million in 2021, with China having nearly 140 million diabetes patients, ranking first worldwide (Ogurtsova et al., 2022; Sun et al., 2021). While current therapies for diabetes mainly rely on medicines, traditional medicine in many countries has also recognized the positive effects of local plant resources in diabetes treatment (Emordi et al., 2016; Li et al., 2004). It has been found that more than 30% of diabetic people use complementary and alternative medicine (Bell et al., 2006). In addition, herbal medicine, natural products, and dietary supplements have demonstrated significant hypoglycemic effects. Consequently, the development of effective substitutes for hypoglycemic drugs, such as functional foods, dietary components, and supplements, has gained increasing attention in the fields of medicine and food research.

*Momordica charantia* L. (*M. charantia*) is a subtropical vegetable widely used in traditional medicine in China, India, Sri Lanka, and Central American countries, known for its antidiabetic effects (Abascal & Yarnell, 2005; Giovannini et al., 2016; Xie et al., 2011), antioxidant activities (Lu et al., 2012; Poovitha et al., 2017), ability to manage body weight (Nerurkar et al., 2010), and potential as a cancer antagonist (Raina et al., 2016). Oxidative stress is a risk factor for diabetes‐related complications, and an increase in antioxidant activities will be beneficial to the management of the complications (Deshaware et al., 2017).

Among the various bioactive components of *M. charantia*, saponins are considered major hypoglycemic agents, with more than 30 saponins demonstrating antidiabetic potential (Deng et al., 2022). Keller et al. confirmed that a saponin‐rich fraction and isolated compounds from *M. charantia* stimulated insulin secretion in MIN6 β‐cells (Keller et al., 2011). In a study, *M. charantia* extracts (with a total content of three triterpenes at 0.69%) were shown to ameliorate the diabetic and hyperlipidemic state in high‐fat‐fed mice by regulating hepatic Phosphoenolpyruvate carboxykinase and AMP‐activated protein kinase (AMPK) phosphorylation (Shih et al., 2014). Additionally, an in vitro experiment revealed that five cucurbitane glycosides from *M. charantia* promoted GLUT4 translocation to the cell membrane, accompanied by increased AMPK activity in L6 myotubes and 3T3‐L1 adipocytes (Tan et al., 2008). Furthermore, a series of cucurbitane glycosides derived from *M. charantia* fruits exhibited potent antihyperglycemic activities, as evaluated by their glucose uptake effects on C2Cl2 myoblast cells (Zhang et al., 2014).

Although numerous published studies have demonstrated the antidiabetic activity of saponin extracts and compounds from *M. charantia*, most of these studies have focused on specific pathways. Consequently, there remains a lack of comprehensive understanding regarding the overall effects of *M. charantia* saponins (MCS) on antidiabetic activity, and a clear mechanism involving multiple perspectives has yet to be defined.

In the current study, the antidiabetic activity of total saponins from *M. charantia* was investigated using streptozotocin‐induced diabetic mice through multiple approaches. These approaches encompassed the examination of glucose and lipid metabolism, antioxidant effects, inhibition of intestinal alpha‐glucosidase activity, and protective effects on pancreatic islets.

## 2 MATERIALS AND METHODS

### 2.1 Preparation of saponins from *M. charantia*

*M. charantia* (variety: “Youlv No.1303”) was provided by the Vegetable Research Institute of Guangdong Academy of Agricultural Sciences. Saponins were extracted from *M. charantia* according to the method developed by Deng et al. (2022) with some modifications (Deng et al., 2022). Briefly, seeded *M. charantia* fruits were dried at 65°C for 20 h and pulverized to 40 mesh. The *M. charantia* powder was extracted thrice with methanol (five times in volume; 1‐h washing cycle) at 25°C and concentrated with a rotary evaporator (Eyela N‐1100; Eyela) at 55°C. The concentrated extract was redissolved in methanol and purified using MCI gel. The elution solvent was 600 mL of 25% methanol (aqueous solution), 1200 mL of 85% methanol (water solution), and 600 mL of acetone. The 85% methanol–water eluent was concentrated at 55°C under reduced pressure. The concentrated extract was dried in a vacuum freeze dryer (FDU‐2110; Eyela) to obtain MCS. The yield of MCS was 1.61%.

### 2.2 HPLC analysis of saponin composition of MCS

The total saponin content of MCS was 55.39%, which was determined following the vanillin–perchloric acid method. Saponin composition was analyzed using HPLC (Agilent Technologies 1200, USA) technique. Saponin standard samples were provided by Professor Minghua Qiu, Kunming Institute of Botany, Chinese Academy of Sciences. The compounds used and their purity have been presented: Momorcharaside A (95.759%), Momordicoside A (97.728%), Karaviloside XI (58.086%), Momordicoside F2 (86.041%), Momordicoside K (85.729%), Kuguacin N (94.918%), and (23*E*)‐3*β*,7*β*,25‐trihydroxycucubita‐5,23‐dien‐19‐al (97.976%). Previously reported methods were used for analysis (Deng et al., 2017). Briefly, individual triterpenoid saponin compounds were analyzed using an HPLC system equipped with a ZORBAX SB‐C18, 5 μm analytical column (4.6 × 250 mm). The HPLC analysis condition for Momorcharaside A, Momordicoside A, and karaviloside XI have been presented: column temperature: 30°C, HPLC mobile phase: acetonitrile/water, gradient program: 1–16 min, 24%; 16–20 min, 24%–45%; 20–45 min, 45%–55%; and 45–60 min, 55%–80%; flow rate: 1.0 mL/min. The compounds were detected at 203 and 238 nm. The sample injection volume was set at 20 μL. The analytical condition for Momordicoside F2, Momordicoside K, Kuguacin N, and (23*E*)‐3*β*,7*β*,25‐trihydroxycucubita‐5,23‐dien‐19‐al have been presented: column temperature: 30°C, mobile phase: acetonitrile/water; gradient program: 1–50 min, 40%; 50–68 min, 40%–50%; 68–88 min, 50%–75%; flow rate: 1.0 mL/min. The compounds were detected at 203 nm. The sample injection volume was set at 20 μL.

### 2.3 Experimental animals and grouping

Forty‐eight male Kunming mice (20 ± 2 g) were purchased from the Laboratory Animal Center of Southern Medical University (Certificate SCXK 2016‐0041). The animals were kept under standard environmental conditions of 12 h/day (08:00–20:00) at 25 ± 2°C. The mice were allowed to acclimatize for 1 week to the laboratory conditions before the experiment. The procedures to induce diabetic mice models by Pan et al. (2017), Wang et al. (2019), and Jang et al. (2009) were adopted with modifications (Jang et al., 2009; Pan et al., 2017; Wang et al., 2019). Briefly, all mice, except in the normal control, were fasted for 12 h and intraperitoneally injected (i.p) with streptozotocin (80 mg/kg; Sigma) dissolved in ice‐cold citrate buffer (0.1 mol/L, pH 4.2). The normal mice were fasted for the same time and i.p with the same volume of citrate buffer. After 3 days, the mice were allowed to fast for 10 h, and blood samples were obtained following the process of tail vein sampling. The blood glucose levels were monitored with a glucometer (SAFE‐ACCU blood glucose monitoring system, Sannuo). Animals with plasma glucose levels ≥11.0 mmol/L (Du & Zhao, 2012; Jang et al., 2009; Zhang et al., 2023), in addition to polyuria and other diabetic features, were considered to be suffering from diabetes mellitus.

A total of four groups containing 12 mice per group were used for the experiment. Three groups were diabetic, while one group was used as a normal control group (NC, given 0.5% solution of sodium carboxymethylcellulose). The 36 diabetic mice were randomized into three groups: a diabetic control group (DC, given 0.5% solution of sodium carboxymethylcellulose), an MCS low‐dose group (MCSL, given 100 mg/kg of the MCS), and an MCS high‐dose group (MCSH, given 200 mg/kg of the MCS). The doses of the MCS used in the animal experiment were determined as follows: the low dose of 100 mg/kg was equivalent to 6.22 g M. charantia powder/kg (the yield of MCS was 1.61% for M. charantia powder), and the daily intake of 100 mg/kg MCS for a mouse corresponds to a human consumption of approximately 372 g of fresh *M. charantia* fruit per day (taking into account the average human body weight of 60 kg, 1/10 times dose conversion from mice to humans, and the 10% yield of *M. charantia* powder from fresh fruits). MCS was suspended in a 0.5% solution of sodium carboxymethylcellulose. All mice were dosed with gavage daily for 30 days.

All the procedures involving animals followed the guidelines of the national standards outlined in “Laboratory Animal—Requirements of Environment and Housing Facilities” (GB 14925–2010) (General Administration of Quality Supervision, Inspection and Quarantine of China, Standardization Administration of China, 2010).

### 2.4 Estimation of blood glucose levels and body weight

The mice were weighed at the beginning and end of each experimental cycle. The blood samples were collected every 10 days from the tail vein for fasting blood glucose measurement with a glucose assay kit manufactured by Shanghai Rongsheng Biotech Co., Ltd. according to the product manual.

### 2.5 Oral glucose tolerance test

The procedures described by Tanaka et al. (2011) and Guo et al. (2020) were adopted for oral glucose tolerance test with some modifications (Guo et al., 2020; Tanaka et al., 2011). Briefly, on the 30th day, all mice were allowed to fast for 10 h before the mice were treated with 2 g of glucose/kg body weight via intragastric administration. Blood glucose levels were measured using the glucometer at 0, 30, 60, and 120 min after intragastric administration. The areas under the glucose‐time curve (AUC) were calculated as follows:

$$\begin{array}{c} \mathrm{AUC}=½\times \left(\text{blood glucose}\;\mathrm{at}\;0\;\min+\text{blood glucose}\;\mathrm{at}\;30\;\min\right)\times 0.5\\ \;+½\times \left(\text{blood glucose}\;\mathrm{at}\;120\;\min+\text{blood glucose}\;\mathrm{at}\;30\;\min\right)\times 1.5. \end{array}$$

### 2.6 Preparation and analysis of serum samples

The procedure described by Guo et al. (2020) was adopted for the preparation of serum samples with some modifications (Guo et al., 2020). Briefly, blood samples of mice for biochemical assays were obtained from an angular vein on day 31. The blood samples were centrifuged at 4000 rpm for 10 min at 4°C (Sorvall Biofuge Stratos; Thermo Electron). Serum was immediately collected, frozen, and stored at −80°C until further analysis. The assay kits for the determination of total cholesterol (TC), triglyceride (TG), and low‐density lipoprotein cholesterol (LDC‐C) were purchased from Shanghai Kexin Biotechnology Research Institute, China. C‐peptide levels were analyzed using the human C‐peptide radioimmunoassay kit (Beijing North Institute of Biological Technology).

### 2.7 Preparation and analysis of liver tissues

Procedure described by Deng et al. (2017) was adopted for the preparation of liver tissues with some modifications (Deng et al., 2017). Briefly, mice were sacrificed by cervical dislocation at the end of the treatment cycle. Liver tissues were excised and weighed after washing the samples with 0.9% saline. The liver underwent quick‐freezing with liquid nitrogen and stored at −80°C. Tissues (100 mg) were rinsed with 0.9% saline and homogenized in 0.9% saline (nine times the volume used for washing) to prepare the 10% homogenate samples. The liver homogenates were centrifuged for 15 min at 4000 rpm at 4°C. The supernatant was collected and analyzed immediately. The protein content in the supernatant was determined following the Coomassie brilliant blue method. The CAT, SOD, GSH, MDA, HK, and hepatic glycogen contents were tested using assay kits purchased from the Nanjing Jiancheng Bioengineering Institute.

### 2.8 Determination of alpha‐glucosidase activity in the small intestinal mucosa of mice

Procedure described by Ortiz‐Andrade et al. (2007) was adopted with some modifications (Ortiz‐Andrade et al., 2007). Briefly, approximately 10 cm of the small intestine was removed by cutting the intestine across the duodenum's upper end and the ileum's lower end. After washing with cold saline solution (0.9%, w/v, NaCl), the small intestinal mucosa was scraped using glass and weighed. Ice‐cold PBS (66.7 mmol/L, pH 5.6; 3 times the volume used for washing) was added to the small intestinal mucosa, and the sample was homogenized in an ice bath. The homogenates were then centrifuged for 15 min at 4000 rpm at 4°C. The supernatant was collected and analyzed. The protein content in the supernatant was determined following the Coomassie brilliant blue method. The supernatant was diluted with physiological saline to a final concentration of 100 μg total protein/50 μL supernatant.

Fifty microliters of malt sugar solution, sucrose solution, and lactose solution (42 mmol/L for each solution) was added separately to 50 μL of the small intestinal mucosa supernatant for incubation at 37°C for 20 min. Following this, Na_2_CO_3_ (20 μL; 0.2 mol/L) was added to terminate the reaction. The solution after the reaction was centrifuged for 15 min at 4000 rpm. The glucose content (Gs, mmol/L) in the supernatant was determined using a glucose assay kit (Nanjing Jiancheng Bioengineering Institute). The amount of glucose produced by catalyzation by each gram of protein (Gp, mmol/g protein) in the supernatant was calculated as follows:

$$\mathrm{Gp}\;\left(\text{mmol}/g\;\text{protein}\right)=\left(\mathrm{Gs}\times 0.225\right)\times 10.$$

### 2.9 Tissue histology

The procedure described by Xing et al. (2021) was adopted with modifications (Xing et al., 2021). Briefly, the pancreatic tissue harvested from each group was fixed in 4% buffered formalin (pH 7.2) for 24 h. The samples were desiccated and embedded in paraffin. The paraffin section (5 μm) was stained with hematoxylin and eosin (H&E). Each section was examined under a light microscope under conditions of high‐power magnification (×100) for structural changes (DMI3000 B; Leica).

### 2.10 Statistical analyses

All data in this study are expressed as the mean ± SD. The data were evaluated following the one‐way analysis of variance (ANOVA) method using SPSS 13.0. The least significance difference (LSD) method and Dunnett's test were conducted to test the significance of the results.

## 3 RESULTS AND ANALYSIS

### 3.1 Composition and content of MCS

The composition and content of MCS extracted from *M. charantia* are shown in Table 1. Among the seven triterpenoid compounds, the content of Momordicoside K was the highest (11.66 μg/mg), and the content of Kuguacin N was the lowest (0.03 μg/mg). The total content of the seven compounds was 18.24 μg/mg.

**TABLE 1:** Composition and content of *Momordica charantia* saponins.

Triterpenoid compounds	Content (μg/mg)
Momorcharaside A	0.21 ± 0.08
Momordicoside A	0.84 ± 0.09
Karaviloside XI	0.30 ± 0.06
Momordicoside F2	3.45 ± 0.27
Momordicoside K	11.66 ± 0.87
3*β*,7*β*,25‐trihydroxycucurbita‐5,23(*E*)‐dien‐19‐al	1.74 ± 0.22
Kuguacin N	0.03 ± 0.00
Total	18.24 ± 0.15

### 3.2 Effect of MCS on body weight

Table 2 presents a summary of the results obtained from the experiments. The data present the effect of MCS on the body weight of the mice and the fasting blood glucose level. At the end of this experiment, the body weight of the normal control mice increased by 17.16%, which was significantly higher than the body weight of the mice belonging to the other groups. The body weight of the diabetic control group mice significantly decreased (by 11.44%). The body weights corresponding to MCSL and MCSH were increased by 1.01% and 3.52%, respectively. There were no significant weight differences between the two treatment groups. This indicates that MCS helps maintain the body weight of diabetic mice.

**TABLE 2:** Effect of *Momordica charantia* saponins on the body weight of mice and the fasting blood glucose levels.

Group	Weight (g)		Blood glucose (mmol/L)			
	Day 1	Day 31	Day 1	Day 11	Day 21	Day 31
NC	26.75 ± 0.77^a^	31.34 ± 0.75^c^ *	3.54 ± 0.93^a^	3.73 ± 1.08^a^	3.02 ± 0.72^a^	3.51 ± 1.19^a^
DC	28.50 ± 1.32^a^	25.24 ± 1.58^a^	27.90 ± 5.75^b^	30.72 ± 5.06^b^	31.43 ± 4.80^c^	29.19 ± 4.19^c^
MCSL	27.84 ± 1.23^a^	28.12 ± 1.52^b^	28.43 ± 4.88^b^	29.77 ± 5.71^b^	27.91 ± 4.05^c^	24.84 ± 3.03^b^ **
MCSH	27.30 ± 1.05^a^	28.26 ± 1.43^b^	27.75 ± 5.27^b^	26.90 ± 2.83^b^	22.25 ± 4.88^b^	20.40 ± 3.14^b^ **

*Note*: Values are expressed as the mean ± SD (*n* = 12). Different letter superscripts indicate significant differences among groups on the same day (*p* < .05).

Abbreviations: DC, diabetic control; MCSH, diabetic mice treated with *M. charantia* saponins at 200 mg/kg bw; MCSL, diabetic mice treated with *M. charantia* saponins at 100 mg/kg bw; NC, normal control.

* *p* < .05 compared with the initial level of body weight (Day 1) of normal rats.

** *p* < .05 compared with initial level of blood glucose (Day 1) in the same group.

### 3.3 Effect of MCS on blood glucose levels

The results in Table 2 indicated that during the experiment, a significant increase in fasting blood glucose levels was observed in the streptozotocin‐induced diabetic control mice compared to the normal control mice from the first day until the last day. For the normal control group, blood glucose in mice kept largely stable at low levels. For the diabetic mice control group, blood glucose in mice kept rising from day 1 to day 21. However, it decreased a little on day 31 compared to day 21. The reason for the decrease in blood glucose level on day 31 may be related to that the increase in blood glucose with time will reach its limits beyond a certain time period, or the blood glucose levels fluctuated constantly at different times of the day and the blood samples might not be taken exactly at the same time of the days, or with the prolongation of feeding time, self‐regulation functions of some mice may be improved somewhat, which may also lead to a gradual decrease in blood sugar, resulting in such a phenomenon separately or in combination. For the MCS L and MCSH groups, both groups displayed a decreasing trend in fasting blood glucose level as the experiment progressed, with the MCSH group showing a bigger drop than the MCSL group in general, although in most cases the differences were not significant except on day 21. There was no significant reduction in plasma glucose levels in the diabetic mice treated with both the low dose and high dose of MCS compared to the diabetic control (DC) group on day 1 and day 11. However, on day 21, the mice in the MCSH group exhibited a significant reduction in glucose levels (*p* < .05) compared to the DC group. By day 31 of treatment, mice treated with a low dose of MCS and a high dose of MCS demonstrated maximum glucose reduction, with reductions of 12.63% and 26.47%, respectively, compared to the initial levels at the start of the experiment.

### 3.4 Effects of MCS on oral glucose tolerance test

**FIGURE 1:**
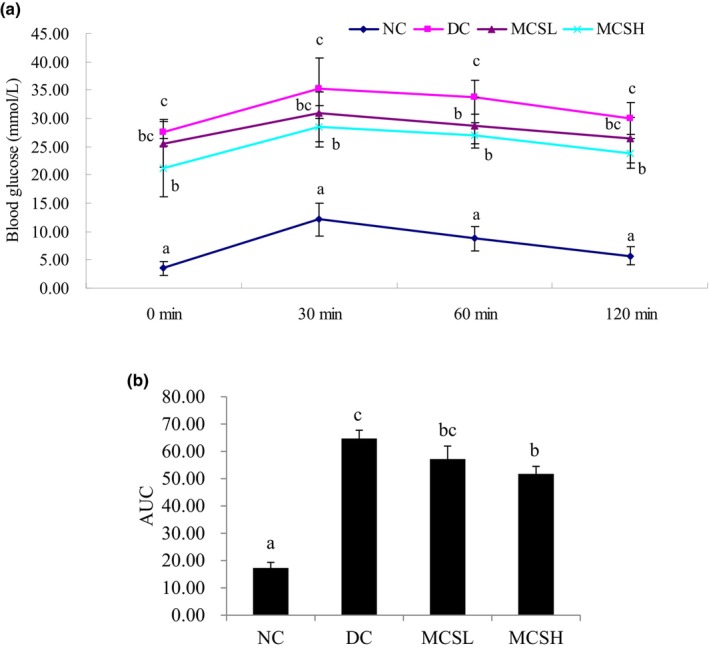
(a) Effects of *Momordica charantia* saponins on the glucose tolerance of diabetic mice. (b) The area under the curve corresponds to postprandial glucose. Values are expressed as the mean ± SD (*n* = 12). Different letters at the same time represent significant differences at *p* < .05. DC, diabetic control; MCSH, diabetic mice treated with *M. charantia* saponins at 200 mg/kg bw; MCSL, diabetic mice treated with *M. charantia* saponins at 100 mg/kg bw; NC, normal control.

Figure 1 illustrates the effect of oral administration of MCS at doses of 100 and 200 mg/kg bw on oral glucose tolerance in diabetic mice, evaluated on day 30 of treatment. After the administration of glucose, the increase in blood sugar levels was inhibited 30 min later in both the MCSL‐ and MCSH‐treated groups. In the MCSH group, the blood sugar level was significantly lower than the diabetic control group (*p* < .05) at 30 min as well as at 120 min. However, the blood sugar levels in the MCSL group did not significantly differ from those of the diabetic control mice at each time point, except at 60 min. The area under the blood glucose curve recorded within 120 min in the diabetic control group was significantly higher than that of the normal control group. In contrast, the area under the curve within 120 min in both the MCSL and MCSH groups was significantly lower than that of the diabetic control group. Based on Figure 1b, it is evident that the sugar tolerance of the diabetic mice improved after the administration of MCS.

### 3.5 Effect of MCS on the microstructure of pancreatic islet

**FIGURE 2:**
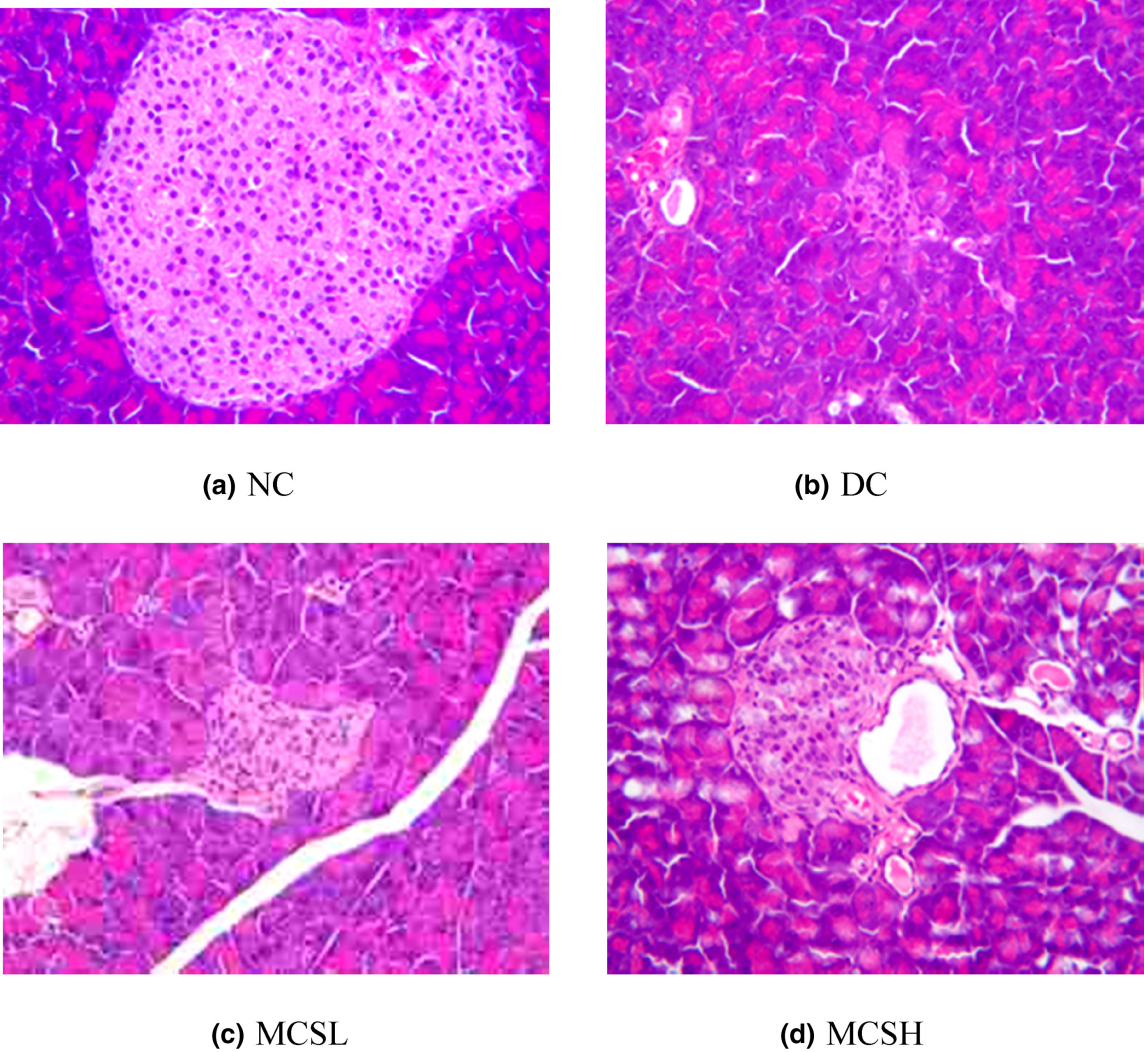
Effects of *Momordica charantia* saponins on the microstructures of the pancreas in diabetic mice (H&E × 100). (a) NC, normal control; normal control mice showed normal intact Langerhans' islets embedded in the acinar cells. (b) DC, diabetic control; diabetic control mice showing severely damaged Langerhans' islets embedded in the acinar cells. (c) MCSL, diabetic mice treated with *M. charantia* saponins at 100 mg/kg bw; results present improved conditions of Langerhans' islets. (d) MCSH, diabetic mice treated with *M. charantia* saponins at 200 mg/kg bw; analysis of diabetic mice revealed the restoration of the normal appearance of Langerhans' islets.

Figure 2 displays the photomicrographs depicting the histological features of the pancreas. The islets of the normal mice exhibit clear and regular boundaries (Figure 2a). The cells within the islets appear well‐organized, with uniform gland size and even distribution. The nuclei were round, with distinct nucleoli, and the cytoplasm was abundant. Figure 2b shows the image of the pancreas of the diabetic control mice. The image reveals sheets of small‐sized glands lined by columnar epithelium. The number of Langerhans islets has decreased, and the structure appears to be lost. The pancreas exhibits complete atrophy, and disintegration and becomes challenging to identify. Figure 2c,d reveal cases where the diabetic mice were treated with MCS at doses of 100 and 200 mg/kg, respectively. The results demonstrate increased islet area and reduced pancreatic fibrosis. The morphology of the islet cells appears better than that of the diabetic control group.

**FIGURE 3:**
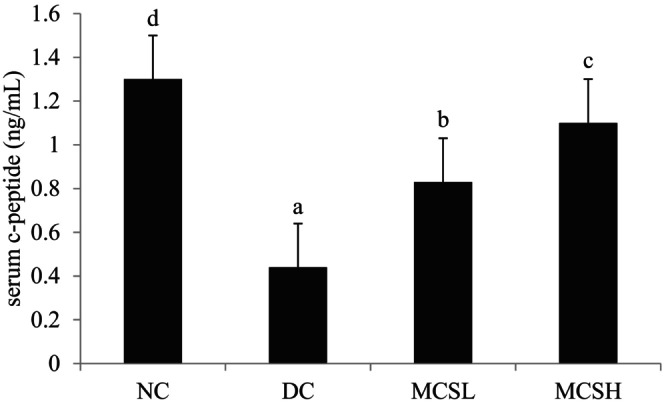
Effects of *Momordica charantia* saponins on C‐peptide in serum in diabetic mice. Values are expressed as the mean ± SD (*n* = 12). Different letters for the same index represent significant differences at *p* < .05. DC, diabetic control; MCSH, diabetic mice treated with *M. charantia* saponins at 200 mg/kg bw; MCSL, diabetic mice treated with *M. charantia* saponins at 100 mg/kg bw; NC, normal control.

Furthermore, a significant increase in C‐peptide levels was observed in the MCS groups compared to the diabetic control (DC) group. This increase showed a dose‐dependent pattern, suggesting that MCS may have the ability to partially restore the impaired insulin‐producing function (Figure 3).

### 3.6 Effect of MCS on hepatic antioxidant capacity

Table 3 presents the summary of the results and the effects of MCS on oxidation parameters. There was a significant decrease (*p* < .05) in the liver CAT, SOD, and GSH levels in the diabetic control mice compared with the normal control. The CAT levels in the diabetic mice treated with MCS were significantly higher (*p* < .05) than those recorded for the diabetic control mice. However, there was no difference between the low‐ and high‐dose groups. The SOD and GSH levels were increased significantly (*p* < .05) only in the diabetic mice treated with MCS at 200 mg/kg bw. There was a significant increase (*p* < .05) in the serum MDA content in the diabetic control mice. The comparison was made with respect to the normal control group. The MDA contents of the diabetic mice treated with MCS decreased significantly (*p* < .05) in a dose‐dependent manner. The comparison was made with respect to the diabetic control group. The results showed that MCS could improve the antioxidant level in the liver of diabetic mice.

**TABLE 3:** Effects of *Momordica charantia* saponins on the antioxidative enzyme activity and MDA levels in the livers of diabetic mice.

Group	CAT (U/mg prot)	SOD (U/mg prot)	GSH (mg/g prot)	MDA (nmol/mg prot)
NC	20.17 ± 1.90^c^	170.46 ± 10.99^d^	2.20 ± 0.19^c^	1.63 ± 0.29^a^
DC	11.24 ± 1.72^a^	120.92 ± 9.92^ab^	1.54 ± 0.14^a^	3.99 ± 0.33^d^
MCSL	15.11 ± 2.11^b^	118.50 ± 6.48^a^	1.66 ± 0.22^ab^	3.49 ± 0.49^c^
MCSH	15.73 ± 0.89^b^	131.59 ± 10.02^b^	1.85 ± 0.18^b^	3.10 ± 0.54^b^

*Note*: Values are expressed as the mean ± SD (*n* = 12). Different letters for the same index represent significant differences at *p* < .05. Abbreviations: DC, diabetic control; MCSH, diabetic mice treated with *M. charantia* saponins at 200 mg/kg bw; MCSL, diabetic mice treated with *M. charantia* saponins at 100 mg/kg bw; NC, normal control.

### 3.7 Effect of MCS on the glycogen content and activity of glucose‐metabolic enzymes in the liver

**FIGURE 4:**
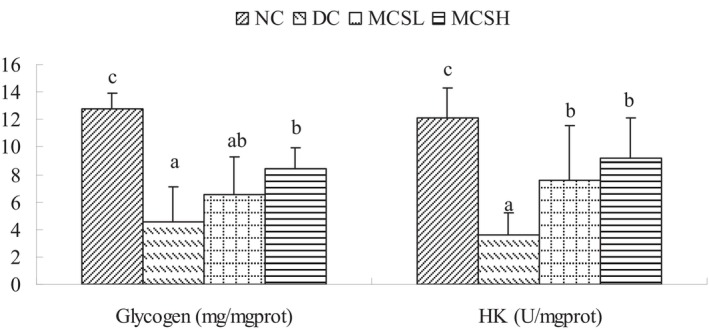
Effect of *Momordica charantia* saponins on the hepatic glycogen and hexokinase levels in the livers of diabetic mice. Values are expressed as the mean ± SD (*n* = 12). Different letters for the same index represent significant differences at *p* < .05. DC, diabetic control; MCSH, diabetic mice treated with *M. charantia* saponins at 200 mg/kg bw; MCSL, diabetic mice treated with *M. charantia* saponins at 100 mg/kg bw; NC, normal control.

Figure 4 presents the levels of hepatic glycogen and hexokinase in liver tissue among the different groups. It was observed that the diabetic mice exhibited significant decreases in hepatic glycogen and hexokinase content. However, after 30 days of treatment, the MCSH group showed a significant increase in hepatic glycogen content (*p* < .05). Additionally, the hexokinase content in the liver was increased in both the MCSL and MCSH groups, with values rising from approximately 3.63 U/mg protein to 7.60 U/mg protein and 9.20 U/mg protein, respectively. These findings indicate that MCS significantly promotes glycogenesis in the liver.

### 3.8 Effect of MCS on the serum lipid profile

**FIGURE 5:**
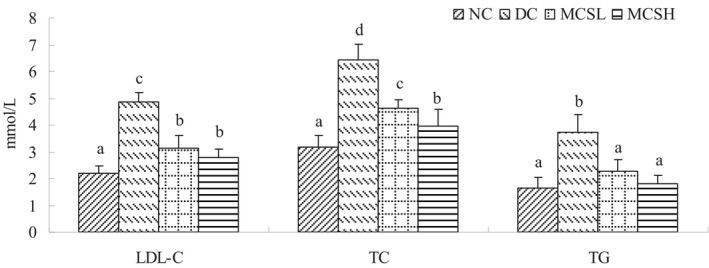
Effect of *Momordica charantia* saponins on the blood lipid level of diabetic mice. Values are expressed as the mean ± SD (*n* = 12). Different letters for the same index represent significant differences at *p* < .05. DC, diabetic control; MCSH, diabetic mice treated with *M. charantia* saponins at 200 mg/kg bw; MCSL, diabetic mice treated with *M. charantia* saponins at 100 mg/kg bw; NC, normal control.

The effects of MCS on the blood lipid profile in diabetic mice are shown in Figure 5. A significant increase in the LDL‐C, TC, and TG levels was observed in the diabetic mice. Following the administration of MCS, the mice exhibited significantly lower lipid levels compared to the diabetic control group (*p* < .05) by the end of the experiment. Particularly noteworthy is the fact that the levels of triglycerides (TG) in the MCS‐treated groups were almost equivalent to those of the mice in the normal control group.

### 3.9 Effect of MCS on the alpha‐glucosidase activity in small intestines

The effects of MCS on sucrase, maltase, and lactase activity in small intestinal mucosa are summarized in Table 4. In the diabetic control group, there was a significant increase in the amount of glucose produced from sucrose, maltose, and lactose per gram of intestinal mucosa homogenate within 20 min compared to the normal control group. There was a significant decrease in the amount of glucose produced from sucrose in the MCSH group (*p* < .05) only compared to the diabetic control group. However, both the MCSL and MCSH groups showed a significant decrease in the amount of glucose produced from maltose and lactose compared to the diabetic control group. Particularly noteworthy is the inhibition of lactase activity in the MCSH group of diabetic mice, which almost matched that of the normal mice. These findings indicate that MCS can inhibit alpha‐glucosidase activity in the small intestines of diabetic mice.

**TABLE 4:** Effects of *Momordica charantia* saponins on the sucrase, maltase, and lactase activity in the small intestines of diabetic mice.

Group	Amount of glucose produced (mmol/20 min*mg protein)		
	Sucrase	Maltase	Lactase
NC	1.65 ± 0.33^a^	11.37 ± 1.64^a^	0.13 ± 0.04^a^
DC	3.46 ± 0.58^c^	15.65 ± 1.84^c^	0.26 ± 0.14^c^
MCSL	3.32 ± 0.56^c^	13.20 ± 2.49^b^	0.19 ± 0.10^b^
MCSH	2.67 ± 0.21^b^	12.90 ± 3.54^b^	0.15 ± 0.08^a^

*Note*: Values are expressed as the mean ± SD (*n* = 12). Different letters for the same index represent significant differences at *p* < .05. Abbreviations: DC, diabetic control; MCSH, diabetic mice treated with *M. charantia* saponins at 200 mg/kg bw; MCSL, diabetic mice treated with *M. charantia* saponins at 100 mg/kg bw; NC, normal control.

## 4 DISCUSSION

STZ, a commonly used drug, is known to induce structural damage and dysfunction of insulin secretion in islet beta cells, making it a popular choice for creating diabetic animal models (Ito et al., 2001). In this study, the STZ‐induced diabetic mice exhibited typical diabetic symptoms. After 30 days of treatment, the diabetic mice experienced weight loss, while their blood sugar levels remained consistently high throughout the experiment. However, significant reductions in blood glucose levels were observed in all the diabetic mice treated with MCS compared to the diabetic control group. These findings indicate that the presence of saponins may have played a key role in enhancing the antidiabetic activity of *M. charantia*.

MCS has been shown to produce a hypoglycemic effect, promote AMPK activity in diabetic mice (Wang et al., 2019), and activate the AMPK phosphorylation pathway in FL83B insulin‐resistant mouse hepatocytes (Cheng et al., 2008). AMPK is a key regulator of energy metabolism. In this study, the hypoglycemic pathway for saponins was evaluated by inhibiting glucose absorption, promoting glycogen synthesis (Figure 4), and regulating lipid metabolism (Figure 5). The increase in hexokinase activity and glycogen content in the liver of diabetic mice (Figure 4) suggested that the administration of the MCS promoted the conversion of glucose to glycogen and inhibited the process of glycogen decomposition. This helped maintain low blood sugar levels. The improvement in glucose metabolism can be attributed to the upregulation of the transcriptional activity of PPARs (Quang et al., 2011). As one of the major hormones involved in glucose metabolism, insulin plays a crucial role in promoting glycogen synthesis. In diabetic mice, the reduced or lack of insulin release leads to low hepatic glycogen levels, as there is inadequate stimulation for the conversion of glucose into glycogen (Sulyman et al., 2016). Furthermore, this study demonstrated that MCS significantly lowered the levels of triglycerides, total cholesterol, and LDL‐cholesterol in the treated diabetic mice (as shown in Figure 5). Although the exact mechanism by which MCS improves lipid metabolism in diabetic mice was not investigated in this study, previous research suggests potential mechanisms. These mechanisms include the inhibition of lipid absorption through the inhibition of pancreatic lipase activity (Oishi et al., 2007) and the reduction of lipid accumulation through the downregulation of PPARγ expression (Popovich et al., 2010). The hypoglycemic effect of MCS observed in this study may be attributed to the activation of AMPK and the inhibition of PPARγ expression. Activation of AMPK regulates glucose metabolism and promotes glucose uptake, while inhibition of PPARγ can help reduce lipid accumulation and improve lipid metabolism.

Saponins have been recognized for influencing carbohydrate metabolism and maintaining glucose homeostasis, primarily by delaying glucose absorption through inhibiting α‐glucosidase activity (Oishi et al., 2007). We found that the activity of α‐glucosidase in the small intestines of the diabetic mice increased significantly. In our study, we observed a significant increase in the activity of α‐glucosidase in the small intestines of diabetic mice, with sucrase, maltase, and lactase activities increasing by 1.10, 0.38, and 1.00 times, respectively, compared to normal mice (as shown in Table 4). However, the administration of MCS resulted in the inhibition of these enzyme activities and a reduction in postprandial hyperglycemia (Table 4). The most plausible mechanism behind this observation is that saponins present in M. *charantia* inhibit the activity of α‐glucosidase by preferentially occupying the enzyme's binding site. This binding effectively blocks the breakdown of carbohydrates into individual glucose molecules, thereby controlling postprandial hyperglycemia (Priscilla et al., 2014). Furthermore, it is suggested that MCS may also inhibit the active transport of nutrients, such as D‐glucose, across the intestine. This inhibition occurs by suppressing the production of ATP, which is responsible for the active transport of these molecules (Mahomoodally et al., 2007). By inhibiting nutrient transport, saponins further contribute to regulating glucose levels and managing postprandial hyperglycemia.

Oxidative stress is a prominent factor in the pathophysiology of diabetes (El Barky et al., 2016). Through glucose autoxidation and protein glycosylation, hyperglycemia induces oxidative stress by generating free radicals (Shahidi et al., 2016). The findings of this study are consistent with previous reports by Jia et al., which demonstrated that diabetes reduces the levels of antioxidant molecules such as GSH, SOD, CAT, GPx, and GR in the liver and serum (Jia et al., 2009). Previous research has suggested that saponins exhibit significant antioxidant activity, primarily due to the presence of hydroxyl groups in their nucleus structure (You et al., 2012). In the present study, treatment with MCS resulted in a significant increase in the activities of antioxidant enzymes and a decrease in the concentration of MDA (a marker of oxidative stress) in the liver. These results indicate that saponins possess effective antioxidative properties and can scavenge excess free radicals. This antioxidative effect is beneficial in repairing the damage to pancreatic β cells caused by STZ‐induced oxidative stress. Furthermore, the reparative effects of *M. charantia* and its extracts on pancreatic β cells (specifically HIT‐T15 pancreatic β cells) have been demonstrated in previous studies. These findings further support the role of *M. charantia* saponins in protecting and repairing pancreatic β cells (Xiang et al., 2007). Moreover, saponin‐rich fraction from *M. charantia* was found to effectively stimulate insulin secretion in MIN6 pancreatic β‐cells in a concentration‐dependent manner, but 3*β*,7*β*,25‐trihydroxycucurbita‐5,23(*E*)‐dien‐19‐al, one of the compounds analyzed in this study, was found to be inefficient (Keller et al., 2011).

**FIGURE 6:**
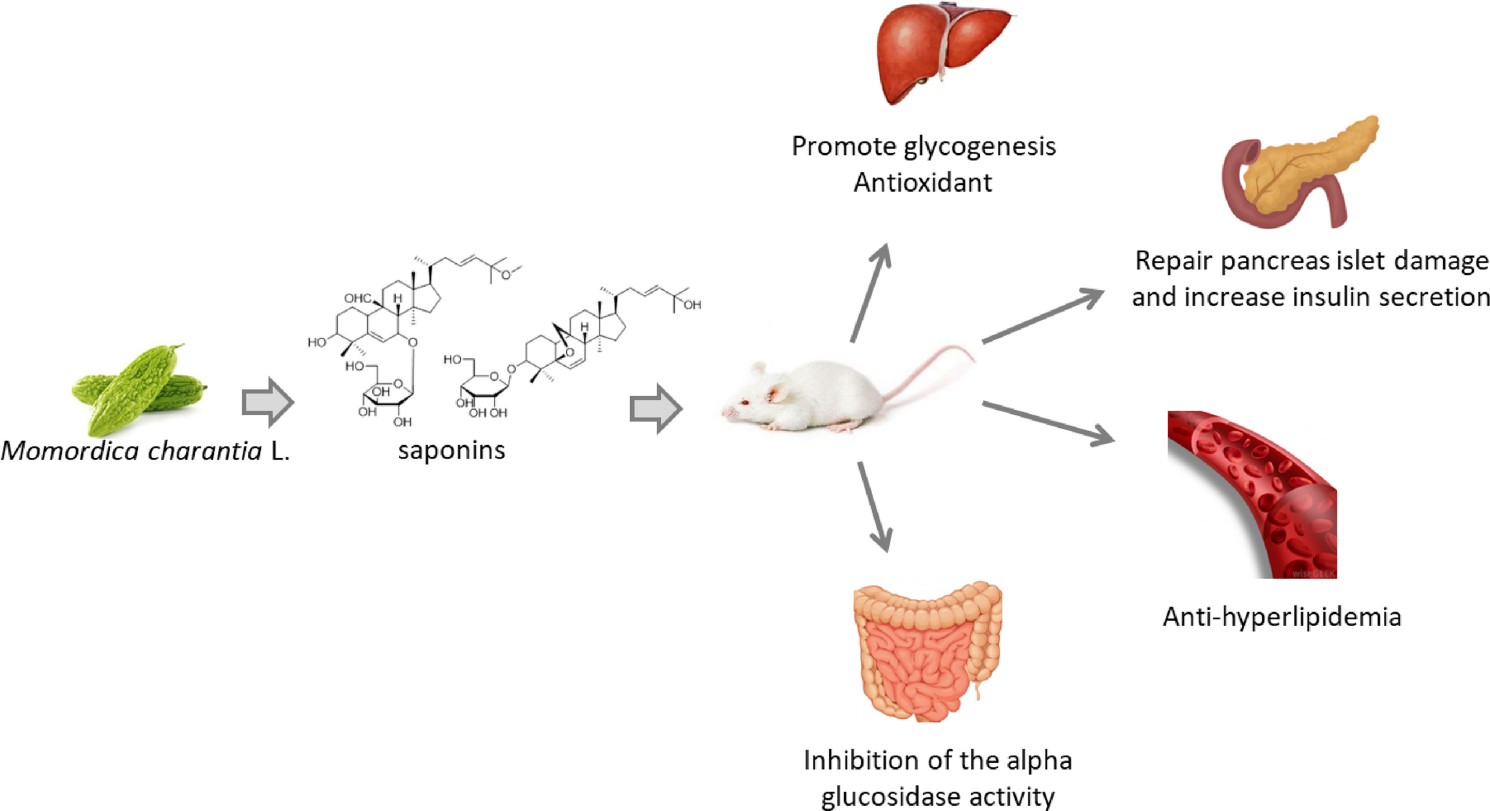
Schematic diagram of the hypoglycemic mechanism of *Momordica charantia* saponins.

It has been reported that more than 150 kinds of saponins have been isolated from *M. charantia*. The structural differences among saponins can lead to variations in their activities. Additionally, the number of glycosyl groups attached to the saponin molecule can significantly influence their absorption, distribution, metabolism, and excretion (Lu et al., 2016). In this study, the contents of seven types of triterpenoid saponins were analyzed. One glycosyl group triterpenoid accounted for more than 84% of the total content of the seven compounds. Among these compounds, Momordicoside K was the most abundant. This high content of triterpenoid saponins with one glycosyl group, particularly Momordicoside K, may explain the observed enhanced activities such as ABTS radical cation scavenging, inhibition of xanthine oxidase (XO) activity, and oxygen radical absorbance capacity (ORAC) values. The antioxidant activity can be attributed to the structural characteristics of cucurbitane‐type triterpenoids with one β‐D‐glucose moiety (Lin et al., 2011). Furthermore, hydroxyl (OH) groups in the saponin structure are responsible for the enhanced antioxidant activity. This property is particularly relevant in the context of diabetes, as it helps to attenuate the formation of reactive oxygen species (ROS), which are known to contribute to oxidative stress in diabetes (El Barky et al., 2016). Karaviloside XI, one of the major triterpenoid compounds found in MCS, has been shown to possess hypoglycemic effects by stimulating AMPK phosphorylation and promoting translocation of glucose transporters‐4 (GLUT4) in both L6 myotubes and 3 T3‐L1 adipocytes (Oishi et al., 2007). This indicates its ability to enhance glucose uptake and utilization in muscle and adipose tissues. Additionally, 3*β*,7*β*,25‐trihydroxycucurbita‐5,23(E)‐dien‐19‐al, another compound present in MCS, has demonstrated significant blood glucose‐lowering effects in alloxan‐induced diabetic mice (Harinantenaina et al., 2006). This compound may contribute to the overall hypoglycemic activity of MCS. These findings suggest that MCS affects glucose metabolism through multiple targets, including the liver, pancreatic beta cells, and small intestines. It regulates various aspects of glycometabolism, such as glucose uptake, utilization, and inhibition of glucose production. The specific mechanisms involve the activation of AMPK, promotion of GLUT4 translocation, and inhibition of α‐glucosidase activity. A schematic diagram illustrating the potential effects of MCS on glycometabolism is presented in Figure 6, which provides a visual representation of the multi‐target regulation of glucose metabolism by MCS.

## 5 CONCLUSION

The findings of this study suggested that the administration of MCS in STZ‐induced diabetic mice resulted in various beneficial effects, including the reduction of fasting blood glucose levels and postprandial hyperglycemia. The inhibitory effects on α‐glucosidase activity contributed to the control of postprandial glucose levels. Additionally, MCS treatment improved the pathological structure and insulin secretion function of the pancreas, as well as serum lipid parameters. The enhancement of hepatic antioxidant enzyme activities and glycogenesis was observed, indicating improved glucose and lipid metabolism. It is concluded that MCS exerts antidiabetic effects by improved hypoglycemic, hypolipidemic, and antioxidant effects, increased hexokinase activity and glycogen synthesis, and enhanced reparative effects on the histological architecture and insulin secretion function of the pancreas. However, it is important to note that further research, including clinical trials, is necessary to validate the effectiveness and safety of MCS in human subjects. Additionally, the underlying molecular mechanisms, specific active components responsible for the observed effects and comparison of effectiveness between MCS and existing drugs should be investigated to better understand the therapeutic potential of MCS in the management of diabetes.

## AUTHOR CONTRIBUTIONS

**Yuanyuan Deng:** Conceptualization (equal); formal analysis (equal); investigation (equal); writing – original draft (equal). **Yan Zhang:** Conceptualization (equal); formal analysis (equal); investigation (equal). **Guang Liu:** Data curation (equal); formal analysis (equal); investigation (equal). **Pengfei Zhou:** Data curation (equal); formal analysis (equal); investigation (equal). **Ping Li:** Data curation (equal); formal analysis (equal); investigation (equal). **Zhihao Zhao:** Data curation (equal); formal analysis (equal); investigation (equal). **Ruifen Zhang:** Investigation (equal); writing – review and editing (equal). **Xiaojun Tang:** Conceptualization (equal); data curation (equal). **Zhangying Wang:** Resources (equal). **Zhencheng Wei:** Conceptualization (equal); funding acquisition (equal); project administration (equal); writing – review and editing (equal). **Mingwei Zhang:** Conceptualization (equal); funding acquisition (equal); project administration (equal); writing – review and editing (equal).

## CONFLICT OF INTEREST STATEMENT

The authors declare that they have no known competing financial interests or personal relationships that could have appeared to influence the work reported in this paper.

## ETHICS STATEMENT

All the procedures involving animals followed the guidelines of the national standards outlined in “Laboratory Animal Requirements of Environment and Housing Facilities” (GB 14925–2010) and experiments were approved by the Ethics Committee for Experimental Animal Care of Sericultural & Agri‐Food Research Institute Guangdong Academy of Agricultural Sciences (Guangzhou, Guangdong, China) and conducted in the animal laboratory (SYXK 2015–0149).

## CONSENT FOR PUBLICATION

All authors have approved the manuscript for publication.

## Data Availability

The data that support the findings of this study are available from the corresponding authors upon reasonable request.
